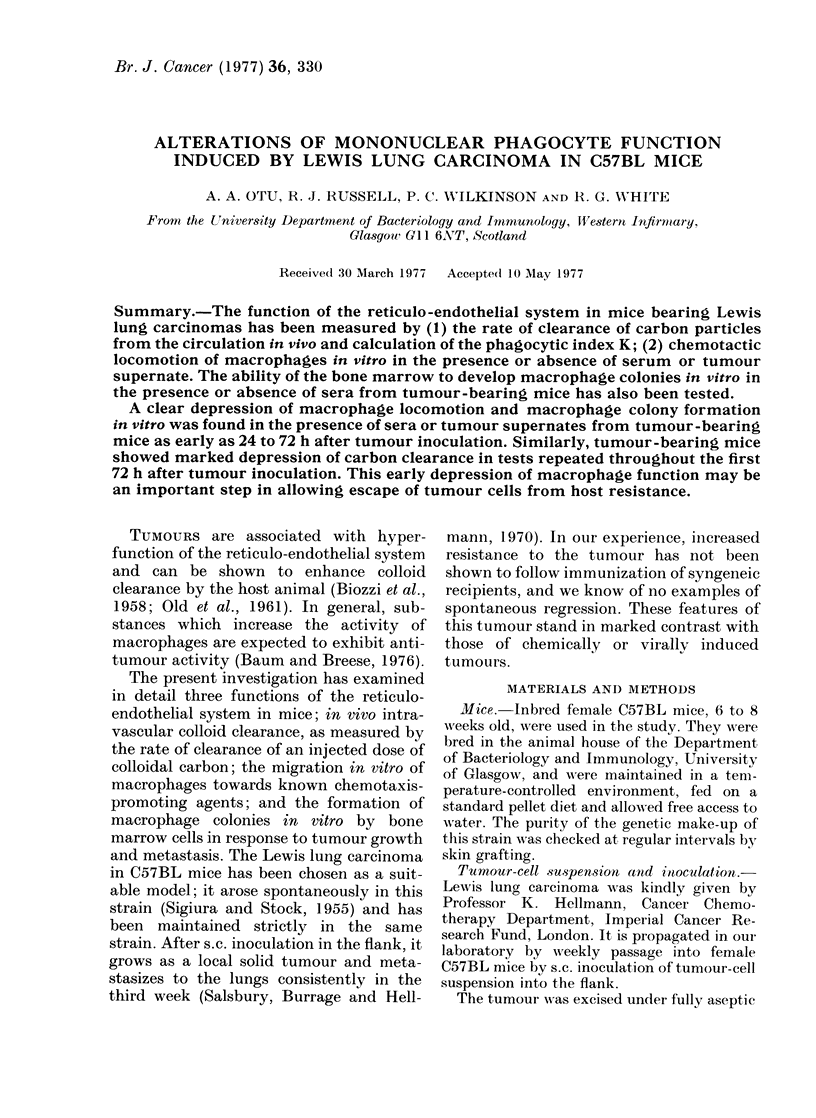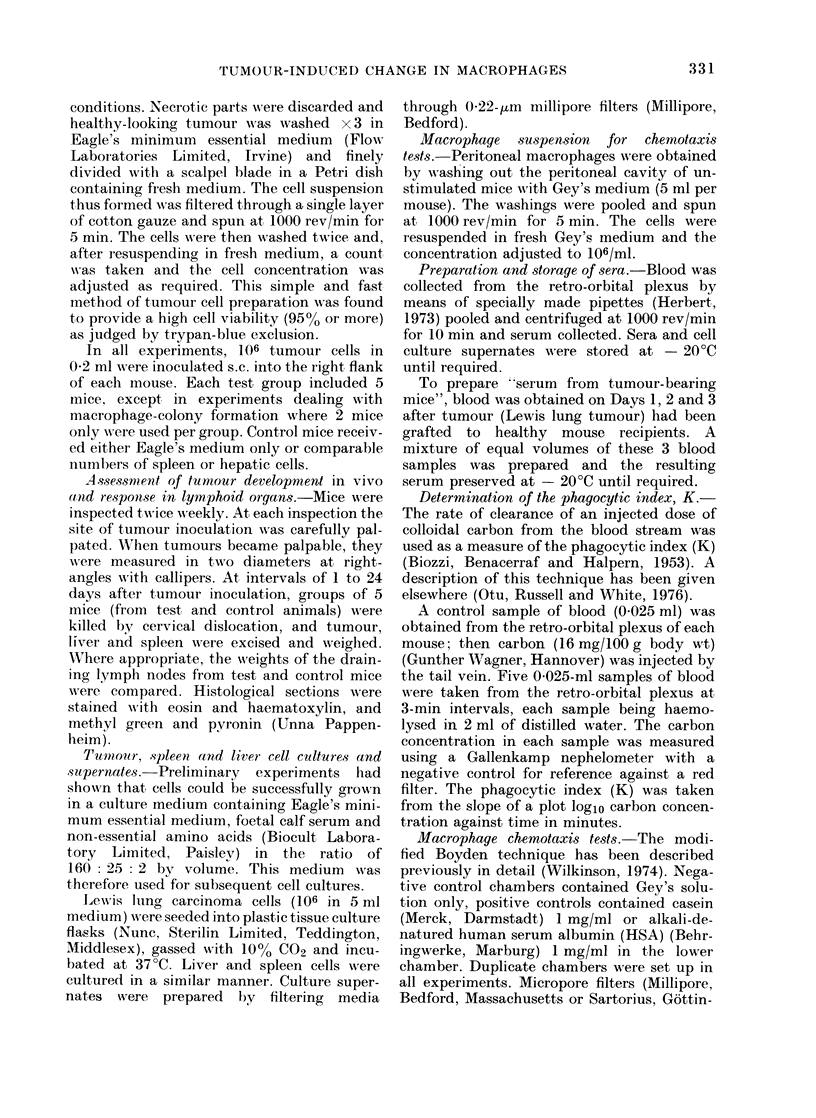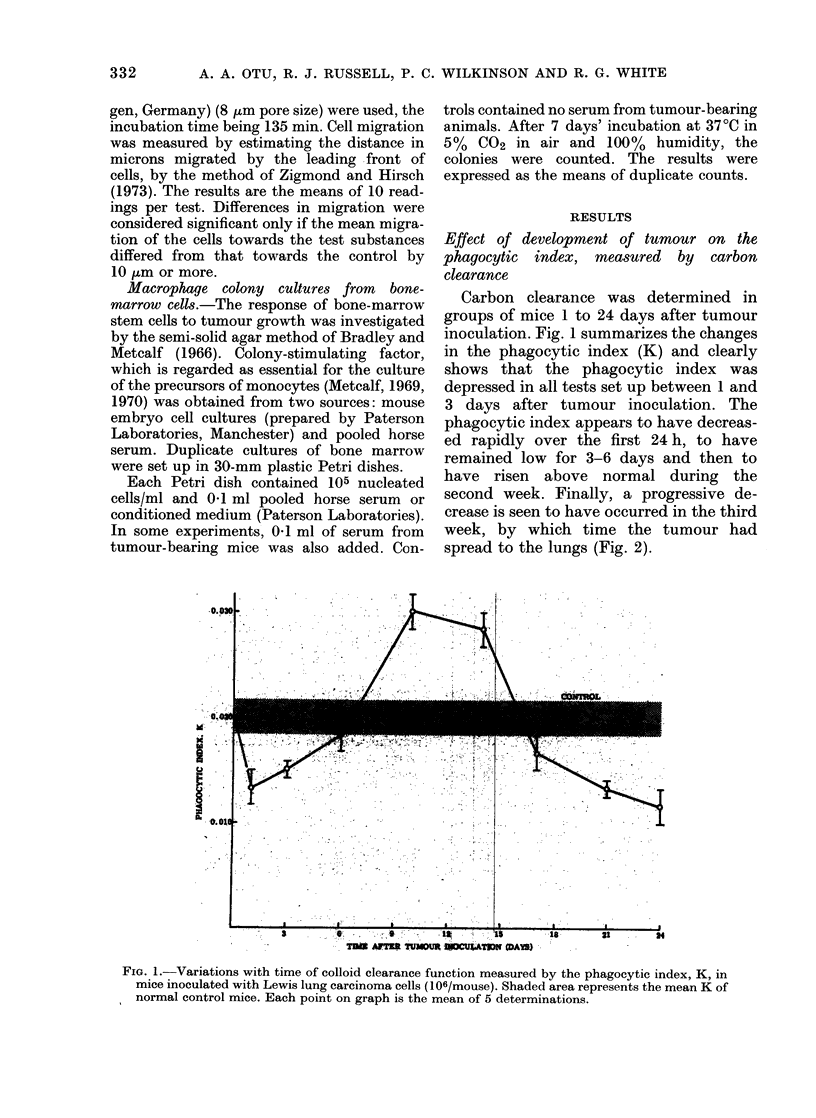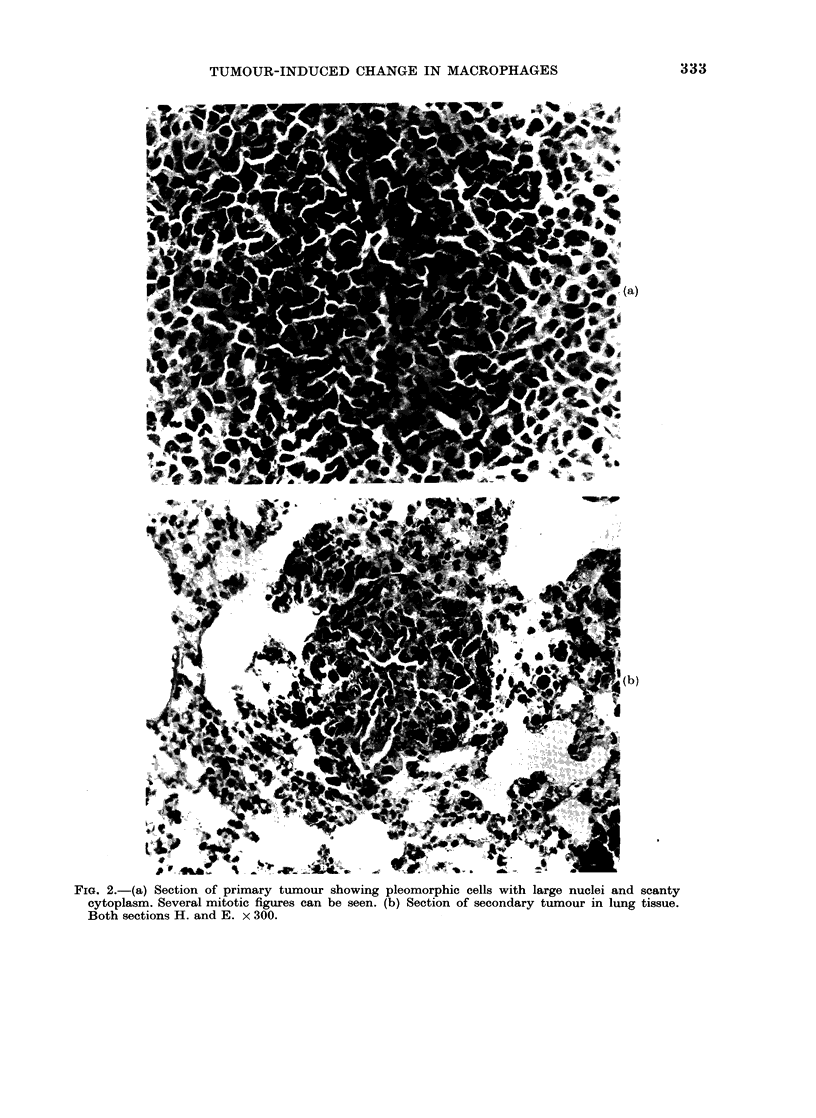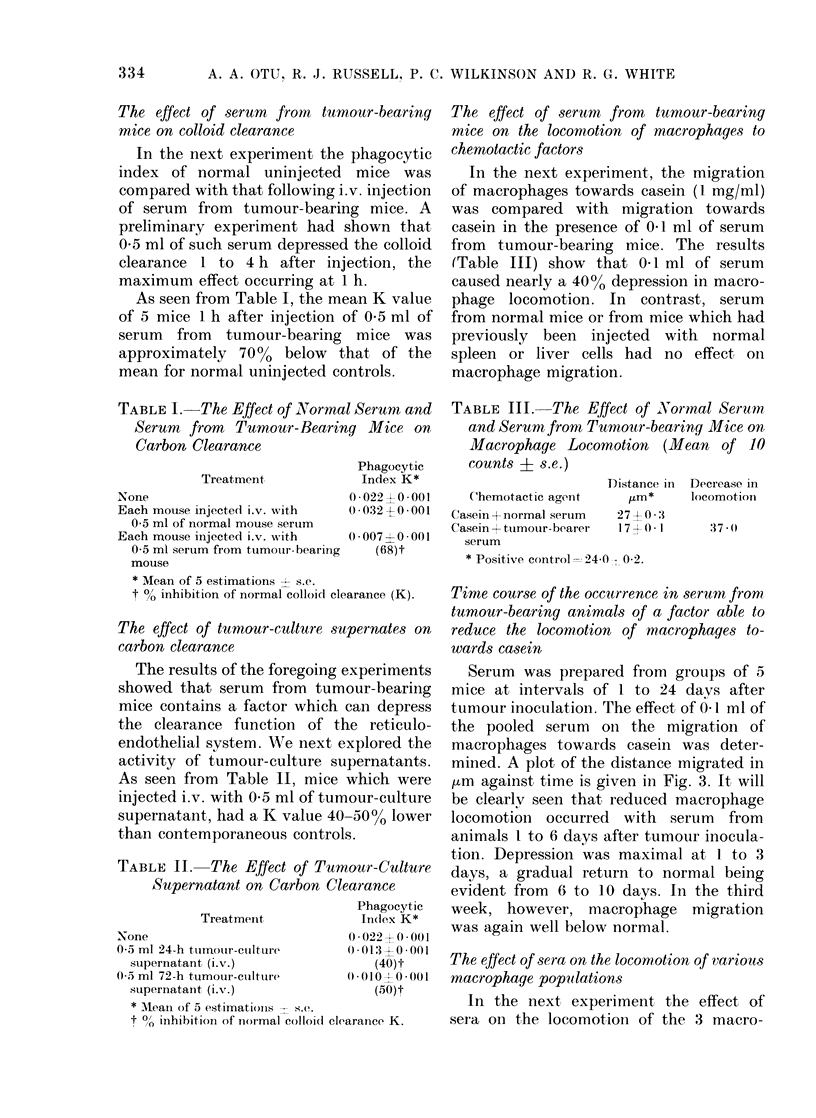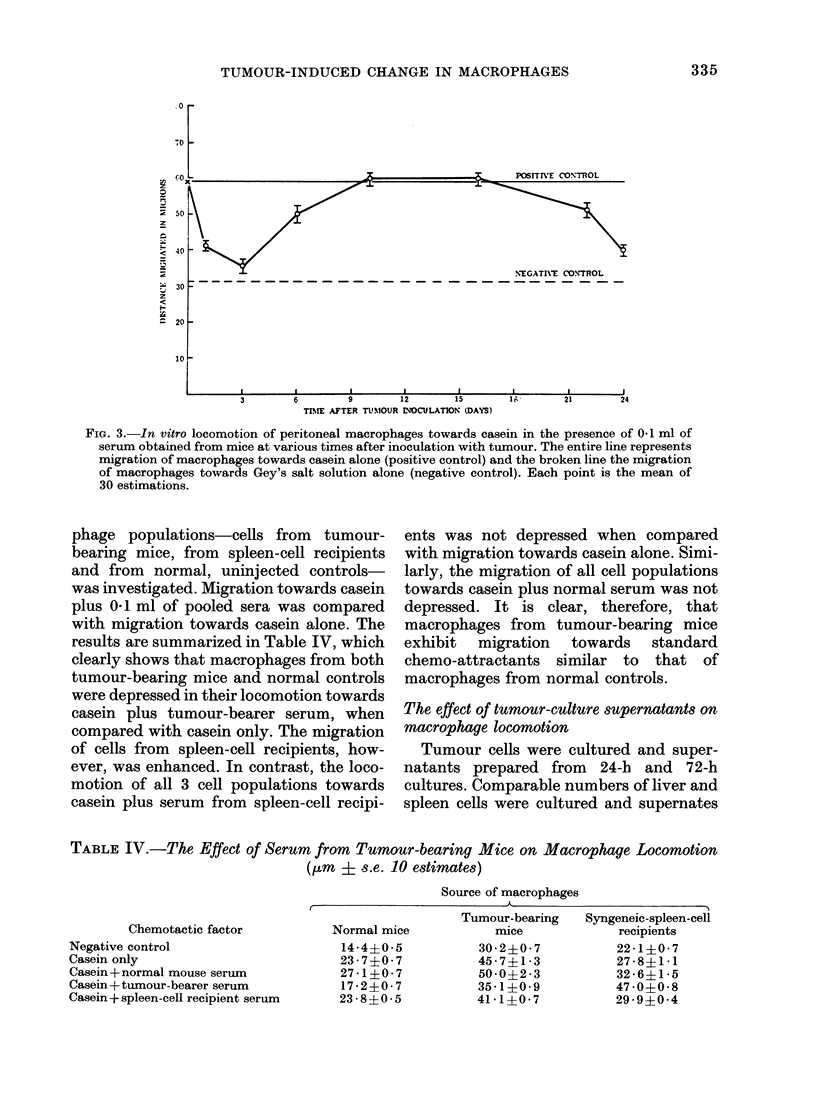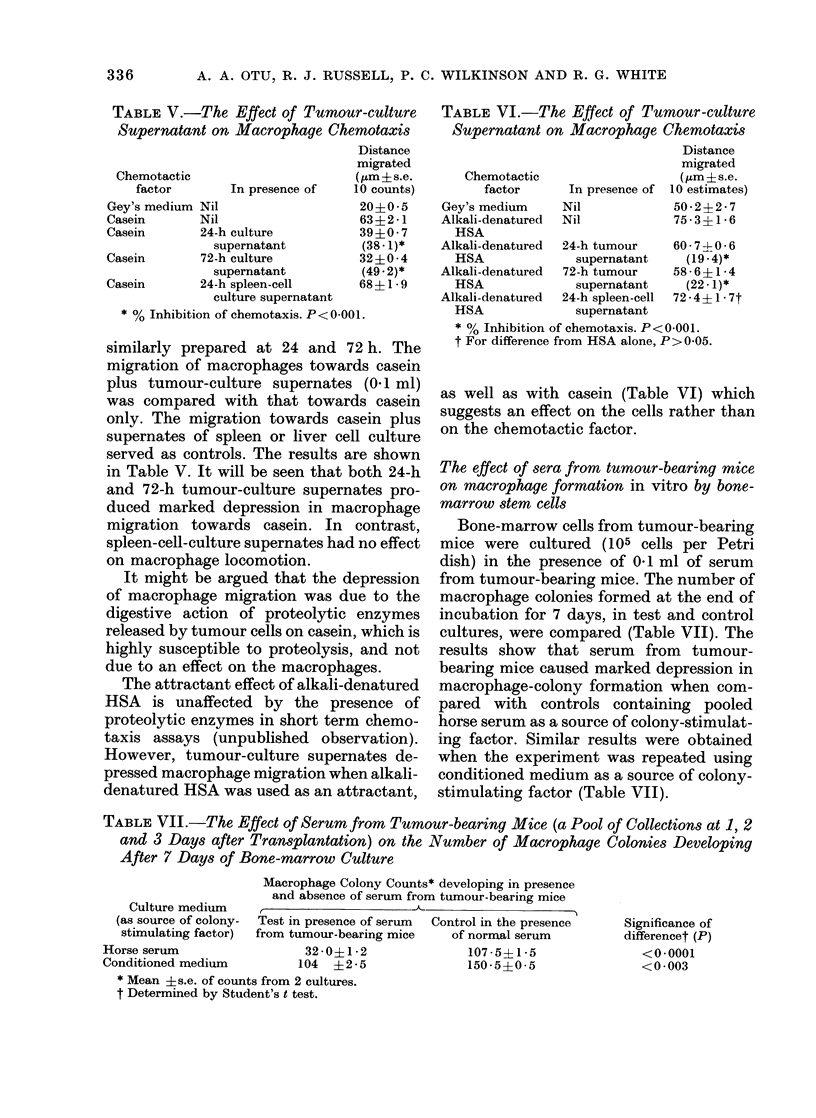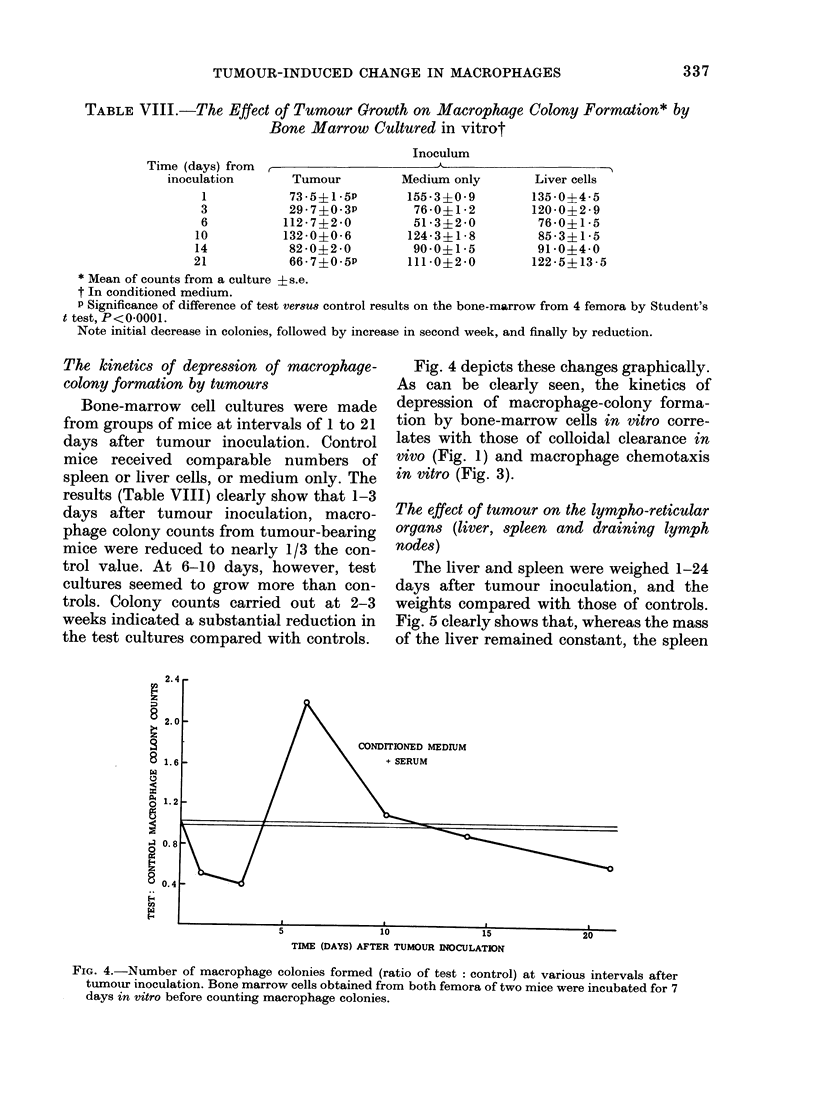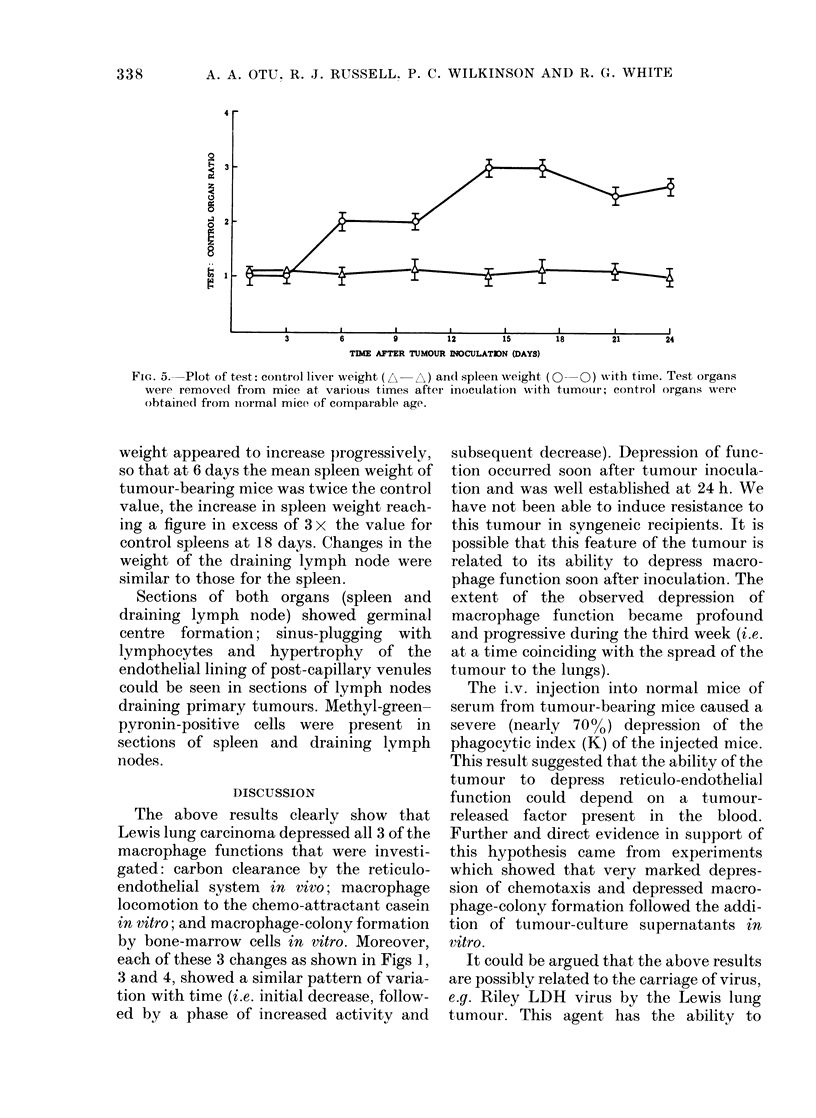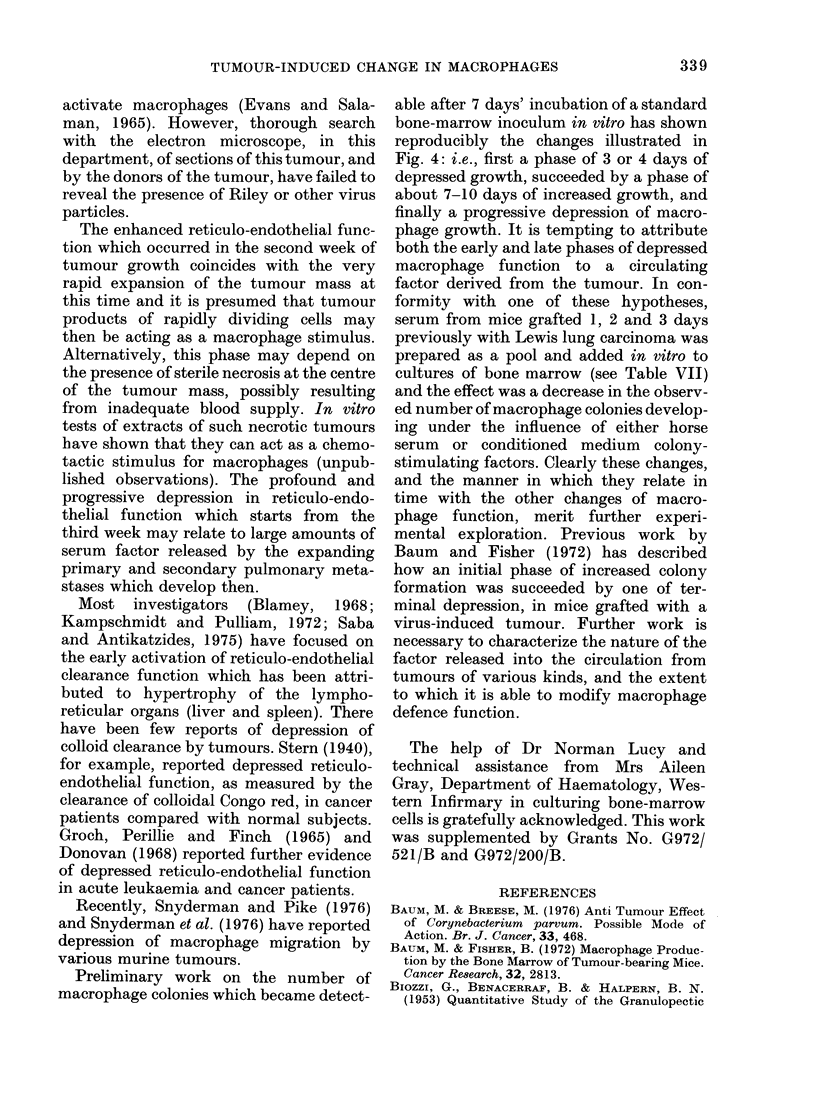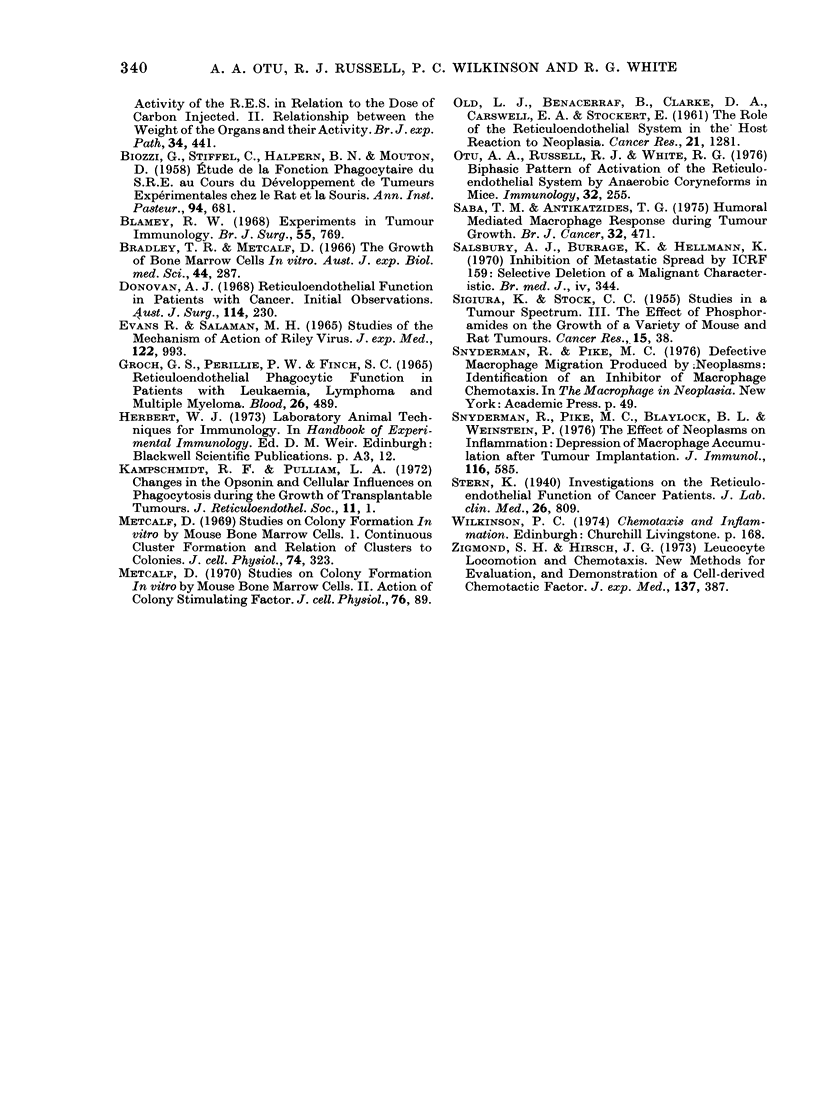# Alterations of mononuclear phagocyte function induced by Lewis lung carcinoma in C57BL mice.

**DOI:** 10.1038/bjc.1977.197

**Published:** 1977-09

**Authors:** A. A. Otu, R. J. Russell, P. C. Wilkinson, R. G. White

## Abstract

**Images:**


*Br. J. Cancer* (1977) **36**, 330

### ALTERATIONS OF MONONUCLEAR PHAGOCYTE FUNCTION INDUCED BY LEWIS LUNG CARCINOMA IN C57BL MICE

A. A. OTU, R. J. RUSSELL, P. C. WILKINSON AND R. G. WHITE

*From the University Department of Bacteriology and Immunology, Western Infirmary, Glasgow G11 6NT, Scotland*

Received 30 March 1977 Accepted 10 May 1977

**Summary.—The function of the reticulo-endothelial system in mice bearing Lewis lung carcinomas has been measured by (1) the rate of clearance of carbon particles from the circulation *in vivo* and calculation of the phagocytic index K; (2) chemotactic locomotion of macrophages *in vitro* in the presence or absence of serum or tumour supernate. The ability of the bone marrow to develop macrophage colonies *in vitro* in the presence or absence of sera from tumour-bearing mice has also been tested.**

**A clear depression of macrophage locomotion and macrophage colony formation *in vitro* was found in the presence of sera or tumour supernates from tumour-bearing mice as early as 24 to 72 h after tumour inoculation. Similarly, tumour-bearing mice showed marked depression of carbon clearance in tests repeated throughout the first 72 h after tumour inoculation. This early depression of macrophage function may be an important step in allowing escape of tumour cells from host resistance.**

TUMOURS are associated with hyperfunction of the reticulo-endothelial system and can be shown to enhance colloid clearance by the host animal (Biozzi *et al.*, 1958; Old *et al.*, 1961). In general, substances which increase the activity of macrophages are expected to exhibit anti-tumour activity (Baum and Breese, 1976).

The present investigation has examined in detail three functions of the reticulo-endothelial system in mice; *in vivo* intravascular colloid clearance, as measured by the rate of clearance of an injected dose of colloidal carbon; the migration *in vitro* of macrophages towards known chemotaxis-promoting agents; and the formation of macrophage colonies *in vitro* by bone marrow cells in response to tumour growth and metastasis. The Lewis lung carcinoma in C57BL mice has been chosen as a suitable model; it arose spontaneously in this strain (Sigiura and Stock, 1955) and has been maintained strictly in the same strain. After s.c. inoculation in the flank, it grows as a local solid tumour and metastasizes to the lungs consistently in the third week (Salsbury, Burrage and Hellmann, 1970). In our experience, increased resistance to the tumour has not been shown to follow immunization of syngeneic recipients, and we know of no examples of spontaneous regression. These features of this tumour stand in marked contrast with those of chemically or virally induced tumours.

### MATERIALS AND METHODS

*Mice.*—Inbred female C57BL mice, 6 to 8 weeks old, were used in the study. They were bred in the animal house of the Department of Bacteriology and Immunology, University of Glasgow, and were maintained in a temperature-controlled environment, fed on a standard pellet diet and allowed free access to water. The purity of the genetic make-up of this strain was checked at regular intervals by skin grafting.

*Tumour-cell suspension and inoculation.*—Lewis lung carcinoma was kindly given by Professor K. Hellmann, Cancer Chemotherapy Department, Imperial Cancer Research Fund, London. It is propagated in our laboratory by weekly passage into female C57BL mice by s.c. inoculation of tumour-cell suspension into the flank.

The tumour was excised under fully aseptic conditions. Necrotic parts were discarded and healthy-looking tumour was washed ×3 in Eagle's minimum essential medium (Flow Laboratories Limited, Irvine) and finely divided with a scalpel blade in a Petri dish containing fresh medium. The cell suspension thus formed was filtered through a single layer of cotton gauze and spun at 1000 rev/min for 5 min. The cells were then washed twice and, after resuspending in fresh medium, a count was taken and the cell concentration was adjusted as required. This simple and fast method of tumour cell preparation was found to provide a high cell viability (95% or more) as judged by trypan-blue exclusion.

In all experiments, $10^6$ tumour cells in 0·2 ml were inoculated s.c. into the right flank of each mouse. Each test group included 5 mice, except in experiments dealing with macrophage-colony formation where 2 mice only were used per group. Control mice received either Eagle's medium only or comparable numbers of spleen or hepatic cells.

*Assessment of tumour development* in vivo *and response in lymphoid organs.*—Mice were inspected twice weekly. At each inspection the site of tumour inoculation was carefully palpated. When tumours became palpable, they were measured in two diameters at right-angles with callipers. At intervals of 1 to 24 days after tumour inoculation, groups of 5 mice (from test and control animals) were killed by cervical dislocation, and tumour, liver and spleen were excised and weighed. Where appropriate, the weights of the draining lymph nodes from test and control mice were compared. Histological sections were stained with eosin and haematoxylin, and methyl green and pyronin (Unna Pappenheim).

*Tumour, spleen and liver cell cultures and supernates.*—Preliminary experiments had shown that cells could be successfully grown in a culture medium containing Eagle's minimum essential medium, foetal calf serum and non-essential amino acids (Biocult Laboratory Limited, Paisley) in the ratio of 160 : 25 : 2 by volume. This medium was therefore used for subsequent cell cultures.

Lewis lung carcinoma cells ($10^6$ in 5 ml medium) were seeded into plastic tissue culture flasks (Nunc, Sterilin Limited, Teddington, Middlesex), gassed with 10% $CO_2$ and incubated at 37°C. Liver and spleen cells were cultured in a similar manner. Culture supernates were prepared by filtering media through 0·22-$\mu$m millipore filters (Millipore, Bedford).

*Macrophage suspension for chemotaxis tests.*—Peritoneal macrophages were obtained by washing out the peritoneal cavity of unstimulated mice with Gey's medium (5 ml per mouse). The washings were pooled and spun at 1000 rev/min for 5 min. The cells were resuspended in fresh Gey's medium and the concentration adjusted to $10^6$/ml.

*Preparation and storage of sera.*—Blood was collected from the retro-orbital plexus by means of specially made pipettes (Herbert, 1973) pooled and centrifuged at 1000 rev/min for 10 min and serum collected. Sera and cell culture supernates were stored at − 20°C until required.

To prepare "serum from tumour-bearing mice", blood was obtained on Days 1, 2 and 3 after tumour (Lewis lung tumour) had been grafted to healthy mouse recipients. A mixture of equal volumes of these 3 blood samples was prepared and the resulting serum preserved at − 20°C until required.

*Determination of the phagocytic index, K.*—The rate of clearance of an injected dose of colloidal carbon from the blood stream was used as a measure of the phagocytic index (K) (Biozzi, Benacerraf and Halpern, 1953). A description of this technique has been given elsewhere (Otu, Russell and White, 1976).

A control sample of blood (0·025 ml) was obtained from the retro-orbital plexus of each mouse; then carbon (16 mg/100 g body wt) (Gunther Wagner, Hannover) was injected by the tail vein. Five 0·025-ml samples of blood were taken from the retro-orbital plexus at 3-min intervals, each sample being haemolysed in 2 ml of distilled water. The carbon concentration in each sample was measured using a Gallenkamp nephelometer with a negative control for reference against a red filter. The phagocytic index (K) was taken from the slope of a plot $\log_{10}$ carbon concentration against time in minutes.

*Macrophage chemotaxis tests.*—The modified Boyden technique has been described previously in detail (Wilkinson, 1974). Negative control chambers contained Gey's solution only, positive controls contained casein (Merck, Darmstadt) 1 mg/ml or alkali-denatured human serum albumin (HSA) (Behringwerke, Marburg) 1 mg/ml in the lower chamber. Duplicate chambers were set up in all experiments. Micropore filters (Millipore, Bedford, Massachusetts or Sartorius, Göttingen, Germany) (8 $\mu$m pore size) were used, the incubation time being 135 min. Cell migration was measured by estimating the distance in microns migrated by the leading front of cells, by the method of Zigmond and Hirsch (1973). The results are the means of 10 readings per test. Differences in migration were considered significant only if the mean migration of the cells towards the test substances differed from that towards the control by 10 $\mu$m or more.

*Macrophage colony cultures from bone-marrow cells.*—The response of bone-marrow stem cells to tumour growth was investigated by the semi-solid agar method of Bradley and Metcalf (1966). Colony-stimulating factor, which is regarded as essential for the culture of the precursors of monocytes (Metcalf, 1969, 1970) was obtained from two sources: mouse embryo cell cultures (prepared by Paterson Laboratories, Manchester) and pooled horse serum. Duplicate cultures of bone marrow were set up in 30-mm plastic Petri dishes.

Each Petri dish contained $10^5$ nucleated cells/ml and 0·1 ml pooled horse serum or conditioned medium (Paterson Laboratories). In some experiments, 0·1 ml of serum from tumour-bearing mice was also added. Controls contained no serum from tumour-bearing animals. After 7 days' incubation at 37 °C in 5% $CO_2$ in air and 100% humidity, the colonies were counted. The results were expressed as the means of duplicate counts.

### RESULTS

*Effect of development of tumour on the phagocytic index, measured by carbon clearance*

Carbon clearance was determined in groups of mice 1 to 24 days after tumour inoculation. Fig. 1 summarizes the changes in the phagocytic index (K) and clearly shows that the phagocytic index was depressed in all tests set up between 1 and 3 days after tumour inoculation. The phagocytic index appears to have decreased rapidly over the first 24 h, to have remained low for 3–6 days and then to have risen above normal during the second week. Finally, a progressive decrease is seen to have occurred in the third week, by which time the tumour had spread to the lungs (Fig. 2).

FIG. 1.—Variations with time of colloid clearance function measured by the phagocytic index, K, in mice inoculated with Lewis lung carcinoma cells ($10^6$/mouse). Shaded area represents the mean K of normal control mice. Each point on graph is the mean of 5 determinations.

FIG. 2.—(a) Section of primary tumour showing pleomorphic cells with large nuclei and scanty cytoplasm. Several mitotic figures can be seen. (b) Section of secondary tumour in lung tissue. Both sections H. and E. $\times 300$.

*The effect of serum from tumour-bearing mice on colloid clearance*

In the next experiment the phagocytic index of normal uninjected mice was compared with that following i.v. injection of serum from tumour-bearing mice. A preliminary experiment had shown that 0·5 ml of such serum depressed the colloid clearance 1 to 4 h after injection, the maximum effect occurring at 1 h.

As seen from Table I, the mean K value of 5 mice 1 h after injection of 0·5 ml of serum from tumour-bearing mice was approximately 70% below that of the mean for normal uninjected controls.

TABLE I.—*The Effect of Normal Serum and Serum from Tumour-Bearing Mice on Carbon Clearance*

| Treatment | Phagocytic Index K* |
|---|---|
| None | 0·022 ± 0·001 |
| Each mouse injected i.v. with 0·5 ml of normal mouse serum | 0·032 ± 0·001 |
| Each mouse injected i.v. with 0·5 ml serum from tumour-bearing mouse | 0·007 ± 0·001 (68)† |

* Mean of 5 estimations ± s.e.
† % inhibition of normal colloid clearance (K).

*The effect of tumour-culture supernates on carbon clearance*

The results of the foregoing experiments showed that serum from tumour-bearing mice contains a factor which can depress the clearance function of the reticulo-endothelial system. We next explored the activity of tumour-culture supernatants. As seen from Table II, mice which were injected i.v. with 0·5 ml of tumour-culture supernatant, had a K value 40–50% lower than contemporaneous controls.

TABLE II.—*The Effect of Tumour-Culture Supernatant on Carbon Clearance*

| Treatment | Phagocytic Index K* |
|---|---|
| None | 0·022 ± 0·001 |
| 0·5 ml 24-h tumour-culture supernatant (i.v.) | 0·013 ± 0·001 (40)† |
| 0·5 ml 72-h tumour-culture supernatant (i.v.) | 0·010 ± 0·001 (50)† |

* Mean of 5 estimations ± s.e.
† % inhibition of normal colloid clearance K.

*The effect of serum from tumour-bearing mice on the locomotion of macrophages to chemotactic factors*

In the next experiment, the migration of macrophages towards casein (1 mg/ml) was compared with migration towards casein in the presence of 0·1 ml of serum from tumour-bearing mice. The results (Table III) show that 0·1 ml of serum caused nearly a 40% depression in macrophage locomotion. In contrast, serum from normal mice or from mice which had previously been injected with normal spleen or liver cells had no effect on macrophage migration.

TABLE III.—*The Effect of Normal Serum and Serum from Tumour-bearing Mice on Macrophage Locomotion (Mean of 10 counts ± s.e.)*

| Chemotactic agent | Distance in μm* | Decrease in locomotion |
|---|---|---|
| Casein + normal serum | 27 ± 0·3 | |
| Casein + tumour-bearer serum | 17 ± 0·1 | 37·0 |

* Positive control = 24·0 ± 0·2.

*Time course of the occurrence in serum from tumour-bearing animals of a factor able to reduce the locomotion of macrophages towards casein*

Serum was prepared from groups of 5 mice at intervals of 1 to 24 days after tumour inoculation. The effect of 0·1 ml of the pooled serum on the migration of macrophages towards casein was determined. A plot of the distance migrated in μm against time is given in Fig. 3. It will be clearly seen that reduced macrophage locomotion occurred with serum from animals 1 to 6 days after tumour inoculation. Depression was maximal at 1 to 3 days, a gradual return to normal being evident from 6 to 10 days. In the third week, however, macrophage migration was again well below normal.

*The effect of sera on the locomotion of various macrophage populations*

In the next experiment the effect of sera on the locomotion of the 3 macro-

FIG. 3.—*In vitro* locomotion of peritoneal macrophages towards casein in the presence of 0·1 ml of serum obtained from mice at various times after inoculation with tumour. The entire line represents migration of macrophages towards casein alone (positive control) and the broken line the migration of macrophages towards Gey's salt solution alone (negative control). Each point is the mean of 30 estimations.

phage populations—cells from tumour-bearing mice, from spleen-cell recipients and from normal, uninjected controls—was investigated. Migration towards casein plus 0·1 ml of pooled sera was compared with migration towards casein alone. The results are summarized in Table IV, which clearly shows that macrophages from both tumour-bearing mice and normal controls were depressed in their locomotion towards casein plus tumour-bearer serum, when compared with casein only. The migration of cells from spleen-cell recipients, however, was enhanced. In contrast, the locomotion of all 3 cell populations towards casein plus serum from spleen-cell recipients was not depressed when compared with migration towards casein alone. Similarly, the migration of all cell populations towards casein plus normal serum was not depressed. It is clear, therefore, that macrophages from tumour-bearing mice exhibit migration towards standard chemo-attractants similar to that of macrophages from normal controls.

*The effect of tumour-culture supernatants on macrophage locomotion*

Tumour cells were cultured and supernatants prepared from 24-h and 72-h cultures. Comparable numbers of liver and spleen cells were cultured and supernates

TABLE IV.—*The Effect of Serum from Tumour-bearing Mice on Macrophage Locomotion ($\mu m$ ± s.e. 10 estimates)*

| Chemotactic factor | Source of macrophages: Normal mice | Tumour-bearing mice | Syngeneic-spleen-cell recipients |
|---|---|---|---|
| Negative control | 14·4±0·5 | 30·2±0·7 | 22·1±0·7 |
| Casein only | 23·7±0·7 | 45·7±1·3 | 27·8±1·1 |
| Casein + normal mouse serum | 27·1±0·7 | 50·0±2·3 | 32·6±1·5 |
| Casein + tumour-bearer serum | 17·2±0·7 | 35·1±0·9 | 47·0±0·8 |
| Casein + spleen-cell recipient serum | 23·8±0·5 | 41·1±0·7 | 29·9±0·4 |

TABLE V.—*The Effect of Tumour-culture Supernatant on Macrophage Chemotaxis*

| Chemotactic factor | In presence of | Distance migrated ($\mu$m $\pm$ s.e. 10 counts) |
|---|---|---|
| Gey's medium | Nil | 20 $\pm$ 0·5 |
| Casein | Nil | 63 $\pm$ 2·1 |
| Casein | 24-h culture supernatant | 39 $\pm$ 0·7 (38·1)* |
| Casein | 72-h culture supernatant | 32 $\pm$ 0·4 (49·2)* |
| Casein | 24-h spleen-cell culture supernatant | 68 $\pm$ 1·9 |

* % Inhibition of chemotaxis. $P < 0·001$.

similarly prepared at 24 and 72 h. The migration of macrophages towards casein plus tumour-culture supernates (0·1 ml) was compared with that towards casein only. The migration towards casein plus supernates of spleen or liver cell culture served as controls. The results are shown in Table V. It will be seen that both 24-h and 72-h tumour-culture supernates produced marked depression in macrophage migration towards casein. In contrast, spleen-cell-culture supernates had no effect on macrophage locomotion.

It might be argued that the depression of macrophage migration was due to the digestive action of proteolytic enzymes released by tumour cells on casein, which is highly susceptible to proteolysis, and not due to an effect on the macrophages.

The attractant effect of alkali-denatured HSA is unaffected by the presence of proteolytic enzymes in short term chemotaxis assays (unpublished observation). However, tumour-culture supernates depressed macrophage migration when alkali-denatured HSA was used as an attractant,

TABLE VI.—*The Effect of Tumour-culture Supernatant on Macrophage Chemotaxis*

| Chemotactic factor | In presence of | Distance migrated ($\mu$m $\pm$ s.e. 10 estimates) |
|---|---|---|
| Gey's medium | Nil | 50·2 $\pm$ 2·7 |
| Alkali-denatured HSA | Nil | 75·3 $\pm$ 1·6 |
| Alkali-denatured HSA | 24-h tumour supernatant | 60·7 $\pm$ 0·6 (19·4)* |
| Alkali-denatured HSA | 72-h tumour supernatant | 58·6 $\pm$ 1·4 (22·1)* |
| Alkali-denatured HSA | 24-h spleen-cell supernatant | 72·4 $\pm$ 1·7† |

* % Inhibition of chemotaxis. $P < 0·001$.
† For difference from HSA alone, $P > 0·05$.

as well as with casein (Table VI) which suggests an effect on the cells rather than on the chemotactic factor.

*The effect of sera from tumour-bearing mice on macrophage formation* in vitro *by bone-marrow stem cells*

Bone-marrow cells from tumour-bearing mice were cultured ($10^5$ cells per Petri dish) in the presence of 0·1 ml of serum from tumour-bearing mice. The number of macrophage colonies formed at the end of incubation for 7 days, in test and control cultures, were compared (Table VII). The results show that serum from tumour-bearing mice caused marked depression in macrophage-colony formation when compared with controls containing pooled horse serum as a source of colony-stimulating factor. Similar results were obtained when the experiment was repeated using conditioned medium as a source of colony-stimulating factor (Table VII).

TABLE VII.—*The Effect of Serum from Tumour-bearing Mice (a Pool of Collections at 1, 2 and 3 Days after Transplantation) on the Number of Macrophage Colonies Developing After 7 Days of Bone-marrow Culture*

| Culture medium (as source of colony-stimulating factor) | Macrophage Colony Counts* developing in presence and absence of serum from tumour-bearing mice: Test in presence of serum from tumour-bearing mice | Control in the presence of normal serum | Significance of difference† ($P$) |
|---|---|---|---|
| Horse serum | 32·0 $\pm$ 1·2 | 107·5 $\pm$ 1·5 | $<0·0001$ |
| Conditioned medium | 104 $\pm$ 2·5 | 150·5 $\pm$ 0·5 | $<0·003$ |

* Mean $\pm$ s.e. of counts from 2 cultures.
† Determined by Student's $t$ test.

TABLE VIII.—*The Effect of Tumour Growth on Macrophage Colony Formation* by Bone Marrow Cultured* in vitro†

| Time (days) from inoculation | Inoculum: Tumour | Medium only | Liver cells |
|---|---|---|---|
| 1 | 73·5±1·5p | 155·3±0·9 | 135·0±4·5 |
| 3 | 29·7±0·3p | 76·0±1·2 | 120·0±2·9 |
| 6 | 112·7±2·0 | 51·3±2·0 | 76·0±1·5 |
| 10 | 132·0±0·6 | 124·3±1·8 | 85·3±1·5 |
| 14 | 82·0±2·0 | 90·0±1·5 | 91·0±4·0 |
| 21 | 66·7±0·5p | 111·0±2·0 | 122·5±13·5 |

* Mean of counts from a culture ±s.e.
† In conditioned medium.
p Significance of difference of test *versus* control results on the bone-marrow from 4 femora by Student's *t* test, $P<0{\cdot}0001$.
Note initial decrease in colonies, followed by increase in second week, and finally by reduction.

*The kinetics of depression of macrophage-colony formation by tumours*

Bone-marrow cell cultures were made from groups of mice at intervals of 1 to 21 days after tumour inoculation. Control mice received comparable numbers of spleen or liver cells, or medium only. The results (Table VIII) clearly show that 1–3 days after tumour inoculation, macrophage colony counts from tumour-bearing mice were reduced to nearly 1/3 the control value. At 6–10 days, however, test cultures seemed to grow more than controls. Colony counts carried out at 2–3 weeks indicated a substantial reduction in the test cultures compared with controls.

Fig. 4 depicts these changes graphically. As can be clearly seen, the kinetics of depression of macrophage-colony formation by bone-marrow cells *in vitro* correlates with those of colloidal clearance *in vivo* (Fig. 1) and macrophage chemotaxis *in vitro* (Fig. 3).

*The effect of tumour on the lympho-reticular organs (liver, spleen and draining lymph nodes)*

The liver and spleen were weighed 1–24 days after tumour inoculation, and the weights compared with those of controls. Fig. 5 clearly shows that, whereas the mass of the liver remained constant, the spleen

FIG. 4.—Number of macrophage colonies formed (ratio of test : control) at various intervals after tumour inoculation. Bone marrow cells obtained from both femora of two mice were incubated for 7 days *in vitro* before counting macrophage colonies.

FIG. 5.—Plot of test: control liver weight (△—△) and spleen weight (○—○) with time. Test organs were removed from mice at various times after inoculation with tumour; control organs were obtained from normal mice of comparable age.

weight appeared to increase progressively, so that at 6 days the mean spleen weight of tumour-bearing mice was twice the control value, the increase in spleen weight reaching a figure in excess of 3× the value for control spleens at 18 days. Changes in the weight of the draining lymph node were similar to those for the spleen.

Sections of both organs (spleen and draining lymph node) showed germinal centre formation; sinus-plugging with lymphocytes and hypertrophy of the endothelial lining of post-capillary venules could be seen in sections of lymph nodes draining primary tumours. Methyl-green–pyronin-positive cells were present in sections of spleen and draining lymph nodes.

### DISCUSSION

The above results clearly show that Lewis lung carcinoma depressed all 3 of the macrophage functions that were investigated: carbon clearance by the reticulo-endothelial system *in vivo*; macrophage locomotion to the chemo-attractant casein *in vitro*; and macrophage-colony formation by bone-marrow cells *in vitro*. Moreover, each of these 3 changes as shown in Figs 1, 3 and 4, showed a similar pattern of variation with time (*i.e.* initial decrease, followed by a phase of increased activity and subsequent decrease). Depression of function occurred soon after tumour inoculation and was well established at 24 h. We have not been able to induce resistance to this tumour in syngeneic recipients. It is possible that this feature of the tumour is related to its ability to depress macrophage function soon after inoculation. The extent of the observed depression of macrophage function became profound and progressive during the third week (*i.e.* at a time coinciding with the spread of the tumour to the lungs).

The i.v. injection into normal mice of serum from tumour-bearing mice caused a severe (nearly 70%) depression of the phagocytic index (K) of the injected mice. This result suggested that the ability of the tumour to depress reticulo-endothelial function could depend on a tumour-released factor present in the blood. Further and direct evidence in support of this hypothesis came from experiments which showed that very marked depression of chemotaxis and depressed macrophage-colony formation followed the addition of tumour-culture supernatants *in vitro*.

It could be argued that the above results are possibly related to the carriage of virus, *e.g.* Riley LDH virus by the Lewis lung tumour. This agent has the ability to activate macrophages (Evans and Salaman, 1965). However, thorough search with the electron microscope, in this department, of sections of this tumour, and by the donors of the tumour, have failed to reveal the presence of Riley or other virus particles.

The enhanced reticulo-endothelial function which occurred in the second week of tumour growth coincides with the very rapid expansion of the tumour mass at this time and it is presumed that tumour products of rapidly dividing cells may then be acting as a macrophage stimulus. Alternatively, this phase may depend on the presence of sterile necrosis at the centre of the tumour mass, possibly resulting from inadequate blood supply. *In vitro* tests of extracts of such necrotic tumours have shown that they can act as a chemotactic stimulus for macrophages (unpublished observations). The profound and progressive depression in reticulo-endothelial function which starts from the third week may relate to large amounts of serum factor released by the expanding primary and secondary pulmonary metastases which develop then.

Most investigators (Blamey, 1968; Kampschmidt and Pulliam, 1972; Saba and Antikatzides, 1975) have focused on the early activation of reticulo-endothelial clearance function which has been attributed to hypertrophy of the lymphoreticular organs (liver and spleen). There have been few reports of depression of colloid clearance by tumours. Stern (1940), for example, reported depressed reticulo-endothelial function, as measured by the clearance of colloidal Congo red, in cancer patients compared with normal subjects. Groch, Perillie and Finch (1965) and Donovan (1968) reported further evidence of depressed reticulo-endothelial function in acute leukaemia and cancer patients.

Recently, Snyderman and Pike (1976) and Snyderman *et al.* (1976) have reported depression of macrophage migration by various murine tumours.

Preliminary work on the number of macrophage colonies which became detectable after 7 days' incubation of a standard bone-marrow inoculum *in vitro* has shown reproducibly the changes illustrated in Fig. 4: *i.e.*, first a phase of 3 or 4 days of depressed growth, succeeded by a phase of about 7–10 days of increased growth, and finally a progressive depression of macrophage growth. It is tempting to attribute both the early and late phases of depressed macrophage function to a circulating factor derived from the tumour. In conformity with one of these hypotheses, serum from mice grafted 1, 2 and 3 days previously with Lewis lung carcinoma was prepared as a pool and added *in vitro* to cultures of bone marrow (see Table VII) and the effect was a decrease in the observed number of macrophage colonies developing under the influence of either horse serum or conditioned medium colony-stimulating factors. Clearly these changes, and the manner in which they relate in time with the other changes of macrophage function, merit further experimental exploration. Previous work by Baum and Fisher (1972) has described how an initial phase of increased colony formation was succeeded by one of terminal depression, in mice grafted with a virus-induced tumour. Further work is necessary to characterize the nature of the factor released into the circulation from tumours of various kinds, and the extent to which it is able to modify macrophage defence function.

The help of Dr Norman Lucy and technical assistance from Mrs Aileen Gray, Department of Haematology, Western Infirmary in culturing bone-marrow cells is gratefully acknowledged. This work was supplemented by Grants No. G972/521/B and G972/200/B.

### REFERENCES

BAUM, M. & BREESE, M. (1976) Anti Tumour Effect of *Corynebacterium parvum*. Possible Mode of Action. *Br. J. Cancer*, **33**, 468.

BAUM, M. & FISHER, B. (1972) Macrophage Production by the Bone Marrow of Tumour-bearing Mice. *Cancer Research*, **32**, 2813.

BIOZZI, G., BENACERRAF, B. & HALPERN, B. N. (1953) Quantitative Study of the Granulopectic

Activity of the R.E.S. in Relation to the Dose of Carbon Injected. II. Relationship between the Weight of the Organs and their Activity. *Br. J. exp. Path*, **34**, 441.

BIOZZI, G., STIFFEL, C., HALPERN, B. N. & MOUTON, D. (1958) Étude de la Fonction Phagocytaire du S.R.E. au Cours du Développement de Tumeurs Expérimentales chez le Rat et la Souris. *Ann. Inst. Pasteur.*, **94**, 681.

BLAMEY, R. W. (1968) Experiments in Tumour Immunology. *Br. J. Surg.*, **55**, 769.

BRADLEY, T. R. & METCALF, D. (1966) The Growth of Bone Marrow Cells *In vitro*. *Aust. J. exp. Biol. med. Sci.*, **44**, 287.

DONOVAN, A. J. (1968) Reticuloendothelial Function in Patients with Cancer. Initial Observations. *Aust. J. Surg.*, **114**, 230.

EVANS R. & SALAMAN, M. H. (1965) Studies of the Mechanism of Action of Riley Virus. *J. exp. Med.*, **122**, 993.

GROCH, G. S., PERILLIE, P. W. & FINCH, S. C. (1965) Reticuloendothelial Phagocytic Function in Patients with Leukaemia, Lymphoma and Multiple Myeloma. *Blood*, **26**, 489.

HERBERT, W. J. (1973) Laboratory Animal Techniques for Immunology. In *Handbook of Experimental Immunology*. Ed. D. M. Weir. Edinburgh: Blackwell Scientific Publications. p. A3, 12.

KAMPSCHMIDT, R. F. & PULLIAM, L. A. (1972) Changes in the Opsonin and Cellular Influences on Phagocytosis during the Growth of Transplantable Tumours. *J. Reticuloendothel. Soc.*, **11**, 1.

METCALF, D. (1969) Studies on Colony Formation *In vitro* by Mouse Bone Marrow Cells. 1. Continuous Cluster Formation and Relation of Clusters to Colonies. *J. cell. Physiol.*, **74**, 323.

METCALF, D. (1970) Studies on Colony Formation *In vitro* by Mouse Bone Marrow Cells. II. Action of Colony Stimulating Factor. *J. cell. Physiol.*, **76**, 89.

OLD, L. J., BENACERRAF, B., CLARKE, D. A., CARSWELL, E. A. & STOCKERT, E. (1961) The Role of the Reticuloendothelial System in the Host Reaction to Neoplasia. *Cancer Res.*, **21**, 1281.

OTU, A. A., RUSSELL, R. J. & WHITE, R. G. (1976) Biphasic Pattern of Activation of the Reticuloendothelial System by Anaerobic Coryneforms in Mice. *Immunology*, **32**, 255.

SABA, T. M. & ANTIKATZIDES, T. G. (1975) Humoral Mediated Macrophage Response during Tumour Growth. *Br. J. Cancer*, **32**, 471.

SALSBURY, A. J., BURRAGE, K. & HELLMANN, K. (1970) Inhibition of Metastatic Spread by ICRF 159: Selective Deletion of a Malignant Characteristic. *Br. med. J.*, iv, 344.

SIGIURA, K. & STOCK, C. C. (1955) Studies in a Tumour Spectrum. III. The Effect of Phosphoramides on the Growth of a Variety of Mouse and Rat Tumours. *Cancer Res.*, **15**, 38.

SNYDERMAN, R. & PIKE, M. C. (1976) Defective Macrophage Migration Produced by Neoplasms: Identification of an Inhibitor of Macrophage Chemotaxis. In *The Macrophage in Neoplasia*. New York: Academic Press. p. 49.

SNYDERMAN, R., PIKE, M. C., BLAYLOCK, B. L. & WEINSTEIN, P. (1976) The Effect of Neoplasms on Inflammation: Depression of Macrophage Accumulation after Tumour Implantation. *J. Immunol.*, **116**, 585.

STERN, K. (1940) Investigations on the Reticuloendothelial Function of Cancer Patients. *J. Lab. clin. Med.*, **26**, 809.

WILKINSON, P. C. (1974) *Chemotaxis and Inflammation*. Edinburgh: Churchill Livingstone. p. 168.

ZIGMOND, S. H. & HIRSCH, J. G. (1973) Leucocyte Locomotion and Chemotaxis. New Methods for Evaluation, and Demonstration of a Cell-derived Chemotactic Factor. *J. exp. Med.*, **137**, 387.